# Pan-cancer analysis of UBE2T with a focus on prognostic and immunological roles in lung adenocarcinoma

**DOI:** 10.1186/s12931-022-02226-z

**Published:** 2022-11-10

**Authors:** Kui Cao, Xiaodong Ling, Xiangyu Jiang, Jianqun Ma, Jinhong Zhu

**Affiliations:** 1 Department of Clinical Laboratory, Biobank, Harbin Medical University Cancer Hospital, 150 Haping Road, Harbin, 150040 Heilongjiang China; 2 Department of Clinical Oncology, Harbin Medical University Cancer Hospital, 150 Haping Road, Harbin, 150040 Heilongjiang China; 3 Department of Thoracic Surgery, Harbin Medical University Cancer Hospital, 150 Haping Road, Harbin, 150040 Heilongjiang China

**Keywords:** Pan-cancer, UBE2T, Risk signature, Consensus clustering, Tumor immune infiltration, LUAD

## Abstract

**Background:**

Ubiquitin-conjugating enzyme E2 T (UBE2T) is a potential oncogene. However, Pan-cancer analyses of the functional, prognostic and predictive implications of this gene are lacking.

**Methods:**

We first analyzed UBE2T across 33 tumor types in The Cancer Genome Atlas (TCGA) project. We investigated the expression level of UBE2T and its effect on prognosis using the TCGA database. The correlation between UBE2T and cell cycle in pan-cancer was investigated using the single-cell sequencing data in Cancer Single-cell State Atlas (CancerSEA) database. The Weighted Gene Co-expression Network analysis (WGCNA), Univariate Cox and Least absolute shrinkage and selection operator (LASSO) Cox regression models, and receiver operating characteristic (ROC) were applied to assess the prognostic impact of UBE2T-related cell cycle genes (UrCCGs). Furthermore, the consensus clustering (CC) method was adopted to divide TCGA-lung adenocarcinoma (LUAD) patients into subgroups based on UrCCGs. Prognosis, molecular characteristics, and the immune panorama of subgroups were analyzed using Single-sample Gene Set Enrichment Analysis (ssGSEA). Results derived from TCGA-LUAD patients were validated in International Cancer Genome Consortium (ICGC)-LUAD data.

**Results:**

UBE2T is highly expressed and is a prognostic risk factor in most tumors. CancerSEA database analysis revealed that UBE2T was positively associated with the cell cycle in various cancers(r > 0.60, p < 0.001). The risk signature of UrCCGs can reliably predict the prognosis of LUAD (AUC_1 year_ = 0.720, AUC_3 year_ = 0.700, AUC_5 year_ = 0.630). The CC method classified the TCGA-LUAD cohort into 4 UrCCG subtypes (G1–G4). Kaplan–Meier survival analysis demonstrated that G2 and G4 subtypes had worse survival than G3 (Log-rank test P_TCGA training set_ < 0.001, P_ICGC validation set_ < 0.001). A comprehensive analysis of immune infiltrates, immune checkpoints, and immunogenic cell death modulators unveiled different immune landscapes for the four subtypes. High immunophenoscore in G3 and G4 tumors suggested that these two subtypes were immunologically “hot,” tending to respond to immunotherapy compared to G2 subtypes (p < 0.001).

**Conclusions:**

UBE2T is a critical oncogene in many cancers. Moreover, UrCCG classified the LUAD cohort into four subgroups with significantly different survival, molecular features, immune infiltrates, and immunotherapy responses. UBE2T may be a therapeutic target and predictor of prognosis and immunotherapy sensitivity.

**Supplementary Information:**

The online version contains supplementary material available at 10.1186/s12931-022-02226-z.

## Background

To date, targeted therapy has become the first-line therapy for cancer patients harboring corresponding oncogenic driver mutations. However, drug resistance frequently occurs, resulting from de novo drug-resistant mutations, compensatory activation of collateral signaling pathways, or growth dominance of cells with preexistent drug-resistant genetic alterations [1]. In the past few decades, numerous studies have shown that multi-target therapy is superior to single agents in treating a broad spectrum of cancer. Therefore, it is essential to identify more druggable molecular targets to optimize combination therapy strategies and improve cancer management.

Ubiquitin-conjugating enzyme E2 T (UBE2T), also known as HSPC150 or Fanconi anemia group T protein (FANCT), belongs to the ubiquitin–proteasome degradation system. *UBE2T* gene is located in chromosome 1q32.1, encoding a protein product composed of 197 amino acids [2]. UBE2T was initially identified to participate in repairing DNA damage by ubiquitinating FANCL, FANCD2, and FANCI in Fanconi anemia. In recent years, accumulating studies suggest that UBE2T is also involved in the initiation and development of various tumors [3–6]. UBE2T generally catalyzes ubiquitination modification of target molecules to promote tumorigenesis. Many UBE2T substrates have been reported, including p53 [7] and Mule [8] in hepatocellular carcinoma (HCC), BRCA1 in breast cancer [9], FOXO1 in lung cancer [10], and the receptor for activated protein kinase C (RACK1) in gastric cancer (GC) [11]. M435-1279, a novel UBE2T inhibitor, was reported to suppress the Wnt/β-catenin signaling pathway and GC progression [11]. The oncogenic effect of UBE2T also was reported in osteosarcoma, nasopharyngeal carcinoma, prostate cancer, and esophageal cancer [6, 12–15]. However, the implications of UBE2T in many other types of cancers are lacking. In the past decade, the advent of next-generation sequencing (NGS) technologies and the release of The Cancer Genome Atlas (TCGA) datasets made genomic and transcriptomic data on common cancers accessible publicly. Thus, this is ideal for analyzing and unveiling potential biomarkers’ prognostic and predictive values with Pan-cancer analysis in the precision medicine area.

Our study explored UBE2T expression and its clinic relevance across 33 cancer types in the TCGA database. Moreover, by screening malignancy-related cell behaviors through the CancerSEA database, we found that UBE2T was closely associated with the cell cycle. We applied the weighted gene correlation network analysis (WGCNA) and Least absolute shrinkage and selection operator (LASSO) regression algorithm to identify a prognostic signature based on the UBE2T-related cell cycle gene (UrCCGs) in the lung adenocarcinoma (LUAD). Then we performed consensus clustering (CC) analysis with UrCCGs to divide lung adenocarcinoma into four subtypes, followed by validation analysis in the International Cancer Genome Consortium (ICGC) cohort. Each subtype exhibited different clinical, molecular, and cellular characteristics. Finally, we also determined the status of immune infiltration and immunotherapy sensitivity for different subgroups.

## Materials and methods

### Gene expression analysis

We downloaded the RNA-Seq expression data (FPKM format) of 33 different tumors from the TCGA database (https://portal.gdc.cancer.gov/). The gene expression matrix consisting of 10,530 samples and 60,499 genes was obtained (Additional file 7). The genes were annotated using the Gencode (GENCODE.v32) (ftp://ftp.ebi.ac.uk/pub/databases/gencode/Gencode_human/) GTF file and normalized. We determined the expression levels of UBE2T in 33 types of tumors. For some types of tumors without normal or with few normal tissues [e.g., TCGA-ACC and TCGA-DLBC], we used a web-based analysis tool, the Gene Expression Profiling Interactive Analysis (GEPIA) (http://gepia.cancer-pku.cn/) [16], to compare the UBE2T expression between tumor tissues and normal tissues. This website incorporates gene expression data of tumors from TCGA and various normal tissues from the Genotype-Tissue Expression (GTEx) database (https://www.genome.gov/Funded-Programs-Projects/Genotype-Tissue-Expression-Project). The GEPIA included the re-computed TCGA and GTEx data that were processed from corresponding raw RNA-Seq datasets by the UCSC Xena project based on a uniform pipeline [17]. Batch effects resulting from different data resources were eliminated in GEPIA. UBE2T expression in some normal tissues can be found in Additional file 7. Additionally, the UBE2T expression levels in different stages of all TCGA tumors were visualized by the GEPIA. Then, we explored the protein expression level of UBE2T between primary tumors and normal tissues through the UALCAN portal (http://ualcan.path.uab.edu/analysis-prot.html) [18, 19] (Additional file 7). This website provided protein expression profiles for 13 tumors, including breast cancer, ovarian cancer, colon cancer, clear cell renal cell carcinoma, uterine corpus endometrial carcinoma, lung adenocarcinoma, head and neck squamous carcinoma, pancreatic adenocarcinoma, glioblastoma multiforme, hepatocellular carcinoma, prostate adenocarcinoma, gastric cancer, pediatric Brain Cancer. UALCAN collected data from Clinical Proteomic Tumor Analysis Consortium (CPTAC), comprising expression information for around 10,000 proteins. Briefly, protein expression data obtained from the CPTAC were log2 normalized sample-wise. In each type of tumor, every protein’s Z-value for single samples was expressed as standard deviations (SD) from the median of all tumor samples. UALCAN is a comprehensive, user-friendly, and interactive web resource for analyzing cancer OMICS data. It is developed on PERL-CGI with high-quality graphics with the application of javascript and CSS. UALCAN is built to provide graphs and plots illustrating expression profiles and patient survival information for protein-coding [18, 19]. The Human Protein Atlas (HPA, https://www.proteinatlas.org/) was used to explore the subcellular distribution of UBE2T in tumors [20].

### Survival analysis

Clinical data were fetched from the TCGA, including overall survival (OS), disease-specific survival (DSS), progression-free interval (PFI), and disease-free interval (DFI) survival data. Samples without survival information were excluded. (Additional file 7). The UBE2T mRNA expression median was used to divide the patients into high- and low-expression groups. We plotted the Kaplan–Meier survival curves and forest plots of univariate Cox analysis of UBE2T in 33 tumors by using the following R packages: “survival”, “survminer”, and “forestplot”. Univariate Cox regression analysis was also performed to evaluate the association of UBE2T with prognosis. The two methods are different and compensatory. When they produced discrepant results, the results from univariate Cox regression analysis (semi-parametric test) are recommended since its power is generally higher than that of Kaplan–Meier survival (non-parametric test).

### Single-cell data and correlation analysis

We used the Cancer Single-cell State Atlas_7_ (CancerSEA) (http://biocc.hrbmu.edu.cn/CancerSEA/) database to examine the association of UBE2T with 14 primary tumor-related cellular activities, such as cell cycle, proliferation, and angiogenesis [21] (Additional file 7). This database contained only RNA sequencing data of tumor cells. Briefly, gene signatures of the14 function states were compiled by hand from public databases (e.g., HCMDB, Cyclebase, StemMapper) and relevant publications. The scores of functional states for single cancer cells were evaluated from their corresponding phenotypic gene sets by Gene Set Variation Analysis (GSVA), followed by Spearman’s Rank Correlations to determine the correlations between the functional states and *UBE2T* (FDR < 0.05 and correlation > 0.3). The correlations between UBE2T and scores of functional states were visualized using the “ggplot2” package. Our previous publication demonstrated that UBE2T promoted autophagy in LUAD; therefore, we further investigated the functional relevance of UBE2T in LUAD at the single-cell level. We chose EXP0066 (GSE69405) and EXP0067 (GSE85534) (Additional file 7) because only these two single-cell datasets concern LUAD in the CancerSEA database, which contain single-cell RNA sequencing data of lung adenocarcinoma patient-derived cells. We analyzed the correlation between UBE2T and the cellular events in LUAD cells and drew the heat map with “pheatmap” and “ggstatsplot” packages [22, 23]. We determined genes related to UBE2T expression in LUAD by Pearson correlation analysis (p < 0.05, |r > 0.1|), and retrieved a list of 24,250 UBE2T-related genes (Additional file 7). Subsequently, 1,056 genes related to the cell cycle were downloaded from the CancerSEA database (Additional file 7). A Venn diagram using the “VennDiagram” package was used to identify genes shared by the two gene sets (n = 889), which were considered UBE2T-related cell cycle genes (UrCCGs).

### Construction of risk signature with UrCCGs

The WGCNA algorithm is a standard method to evaluate the potential correlation between gene modules and clinical traits [24]. Information on the clinical stage and survival state was used as traits to search for worthwhile co-expression modules associated with stage and prognosis. The gene network and distinguished modules were built using the one-step network construction function of the “WGCNA” R package. Module-trait relationships were evaluated based on the correlation between modules and traits by Pearson’s correlation test, and modules were considered significantly correlated when p ≤ 0.05. Univariate Cox regression analysis was applied to select prognostic genes from 162 overlapping genes (p < 0.001). LASSO Cox analysis was conducted to construct an optimal risk signature ($\mathrm{The risk score} = ANLN*0.141850720503601+ERLIN1*0.0298200395675116+LDHA*0.21493576811564+ORMDL3*|-0.136485417603082|+SERBP1*0.0850088433535292+VDAC1*0.119576428619984+XRCC5*0.110427437278267+ZNF555*|-0.979403992850046|$), with a 10-round cross-validation restricting overfitting. The correlations between the expression of genes in risk signature and immune cell infiltration, including CD8 + T cells, CD4 + T cells, and dendritic cells, were analyzed by the “Gene” portal of the tumor immune estimation resource (TIMER2.0) online tool (http://timer.comp-genomics.org/). The correlation between gene expression and immune infiltration was estimated by the Pearson correlation test (Additional file 7).

### Discovery and validation of the subtypes by the UBE2T-related cell cycle genes

ConsensusClusterPlus is a stratification tool, which uses the consensus clustering (CC) method to identify unsupervised intrinsic groups with similar biological characteristics [25]. This method was used to interrogate whether the expression profile of 889 UBE2T-related cell cycle genes is sufficient to cluster the TCGA-LUAD patients into independent groups with different gene expression patterns. We conducted 500 bootstraps, and each subsample contained 80% of patients in the cohort. Cluster sets ranged from 2 to 9, and the optimal separation was determined by evaluating the consensus matrix and the consensus cumulative distribution function. The subtypes by UrCCGs were validated in an independent International Cancer Genome Consortium (ICGC)-LUAD cohort (https://dcc.icgc.org/) with the same settings (Additional file 7).

### Analysis of tumor behavior states, immune infiltrates, and immune biomarkers

The stromal score, immune score, and tumor purity were compared among different UrCCG subtypes by the ESTIMATE (Estimation of Stromal and Immune cells in Malignant Tumor tissues using Expression data) method (https://bioinformatics.mdanderson.org/estimate/disease.html) [26]. Moreover, all gene sets were acquired from HALLMARK, KEGG pathway, and Gene Ontology databases (Additional file 7). We applied the single-sample Gene Set Enrichment Analysis (ssGSEA) algorithm of the R package “GSVA” to analyze the level of critical functional states of cells and signaling pathways in LUAD by utilizing corresponding gene signatures.

Cellular compositions of immune infiltrates were computed using the metagene approach. We adopted ssGSEA to assess immune cell types from a set of Pan-cancer metagenes for 28 immune cell subpopulations established previously (Additional file 7) [27]. We also retrieved the immunophenoscore (IPS) for LUAD patients from The Cancer Immunome Atlas (TCIA, https://tcia.at/home). The IPS is calculated based on four significant categories of tumor immunogenicity determinants, including effector cells, immunosuppressive cells, major histocompatibility complex (MHC) molecules (antigen processing), and checkpoints/immunomodulators. The IPS, ranging from 0 to 10, was calculated based on the z-score for the expression of related genes (Additional file 7).

### Statistical analysis

All statistical analyses were performed by using R version 4.0.3 (R packages: pheatmap, ggplot2, rms, glmnet, forest, limma, GSVA, survminer, survival ROC). For all analyses. A two-tailed p < 0.05 was regarded as statistical significance if not noted.

## Result

### UBE2T expression analysis in Pan-cancer

**Fig. 1:**
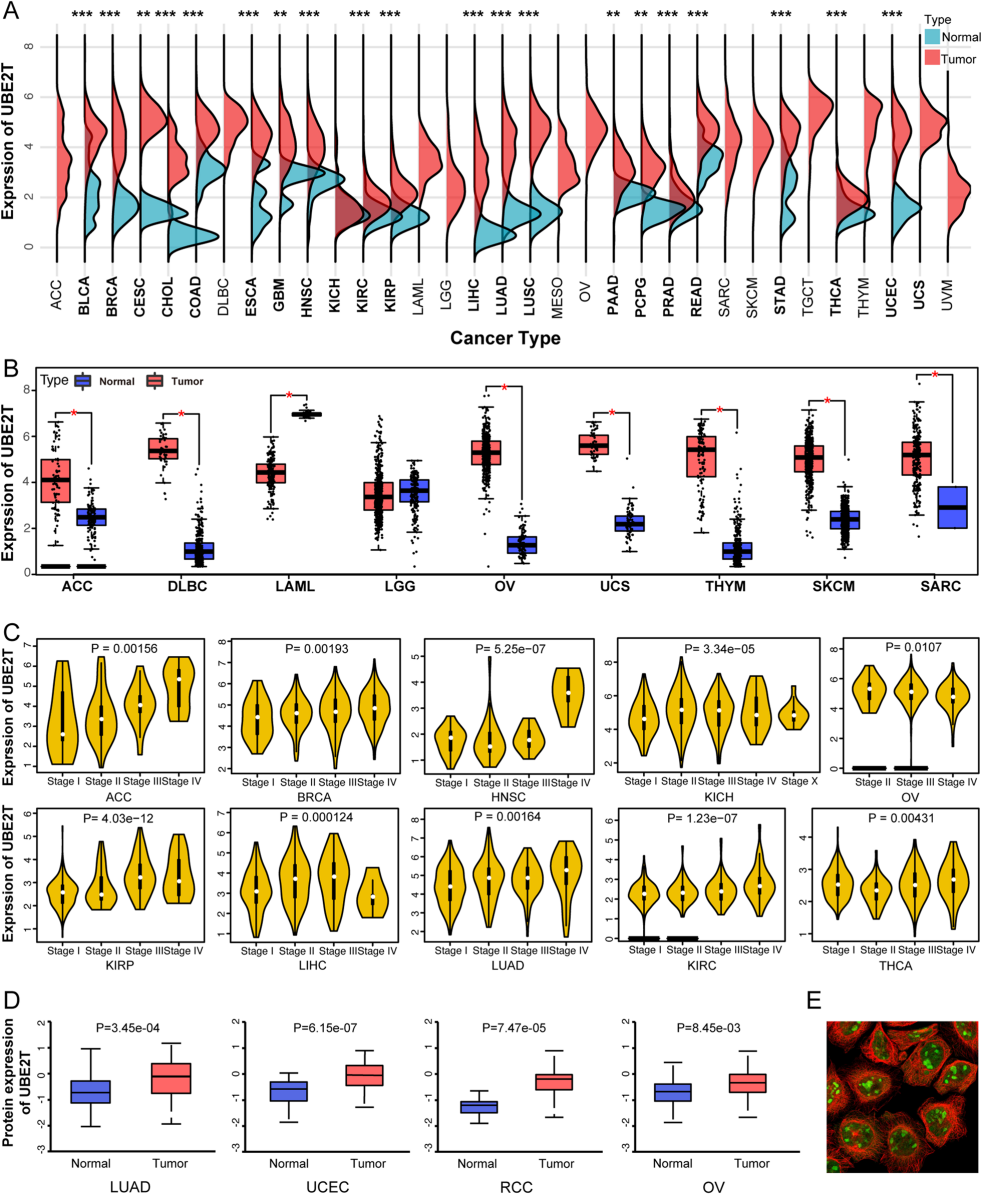
The expression level of the UBE2T gene in different tumors and pathological stages. **A** The expression of the UBE2T gene in 33 different types of tumors and normal tissues in the TCGA database (https://portal.gdc.cancer.gov/). **B** For certain tumors without normal or with few normal tissues (n < 3), the Gene Expression Profiling Interactive Analysis (GEPIA) web tool (http://gepia.cancer-pku.cn/) was used to compare the UBE2T expression between these tumor tissues and the corresponding normal tissues from the Genotype-Tissue Expression (GTEx) database. (https://www.genome.gov/Funded-Programs-Projects/Genotype-Tissue-Expression-Project). **C** Based on the UALCAN database (http://ualcan.path.uab.edu/analysis-prot.html), we compared UBE2T protein expression levels between tumor and respective normal tissues for LUAD, OV, RCC, and UCEC. **D** Using the TCGA data, the expression levels of the UBE2T gene were determined in stages I, II, III, and IV). Log2 (TPM + 1) was applied for the log scale. **E** The subcellular localization of UBE2T in tumor cells was visualized (HPA database) (https://www.proteinatlas.org/). Red and green fluorescence represent microtubules and UBE2T, respectively). *p < 0.05; **p < 0.01; ***p < 0.001

The flowchart of this study is shown in Additional file 1: Fig. S1. We first compared UBE2T expression between cancerous and normal tissues across 33 tumors from the TCGA database (https://portal.gdc.cancer.gov/) (Fig. 1A). For indicated tumor types lacking adequate normal tissues (Fig. 1B), we compared the UBE2T expression between tumor tissues and normal tissues using a web-based analysis tool, GEPIA (http://gepia.cancer-pku.cn/), comprising gene expression in normal tissues from GETX (https://www.genome.gov/Funded-Programs-Projects/Genotype-Tissue-Expression-Project). Overall, UBE2T expression was significantly upregulated in various cancers. Additionally, GEPIA-based analysis showed that UBE2T was differentially expressed in different stages in adrenocortical carcinoma (ACC), invasive breast carcinoma (BRCA), head and neck squamous cell carcinoma (HNSC), kidney chromophobe (KICH), ovarian serous cystadenocarcinoma (OV), kidney renal papillary cell carcinoma (KIRP), liver hepatocellular carcinoma (LIHC), LUAD, kidney renal clear cell carcinoma (KIRC), and thyroid carcinoma (THCA) (Fig. 1C, only significant results shown). The “Protein” module of UALCAN online tool (http://ualcan.path.uab.edu/analysis-prot.html) confirmed that UBE2T protein levels were significantly upregulated in LUAD, uterine corpus endometrial carcinoma (UCEC), renal cell carcinoma (RCC), OV, and BRCA (Fig. 1D, only significant results shown). The HPA database (https://www.proteinatlas.org/) revealed both cytoplasmic and nuclear distribution of UBE2T (Fig. 1E).

### Prognostic implications of UBE2T in Pan-cancer

**Fig. 2:**
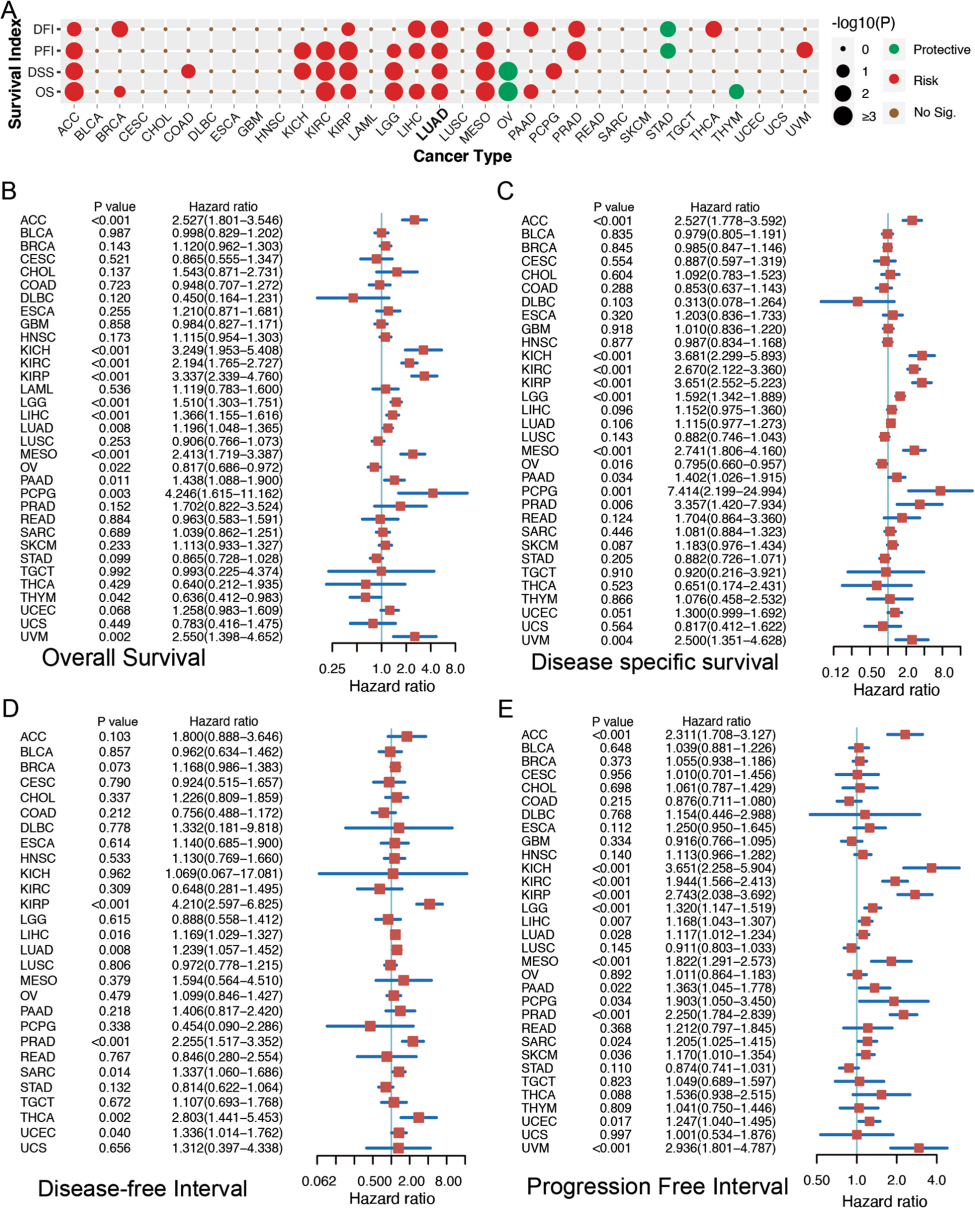
Correlation between UBE2T gene expression and survival of 33 different types of tumors in TCGA database. **A** We used the “survival” and “ggplot2” packages of R software to perform survival analyses regarding UBE2T across 33 different types of tumors, regarding overall survival (OS), Disease-specific Survival (DSS), disease-free Interval (DFI), progression Free Interval (PFI). **B**–**E** The forest plots of univariate Cox regression analysis for OS (**B**), DSS (**C**), DFI (**D**), and PFI (**E**)

We investigated the clinical relevance of UBE2T in the different tumor types using Kaplan–Meier survival analysis (Fig. 2A; Additional file 2: Fig. S2, Additional file 3: Fig. S3, Additional file 4: Fig. S4, Additional file 5: Fig. S5) regarding OS, DSS, DFI, and PFI. Univariate Cox regression analyses were further conducted to assess the associations of UBE2T with prognosis (Fig. 2B–E). The role of UBE2T is tumor-specific. For instance, the high UBE2T levels were associated with poor OS in ACC, BRCA, KIRC, KIRP, brain lower-grade glioma (LGG), LIHC, LUAD, mesothelioma (MESO), but associated with favorable OS in OV (Fig. 2A; Additional file 2: Fig. S2). It is noteworthy that UBE2T was most associated with the survival of LUAD (Fig. 2A). Univariate Cox regression analysis showed that UBE2T expression was a prognostic risk factor of OS in KICH, KIRC, KIRP, LAML, LGG, LUAD, MESO, OV, pancreatic adenocarcinoma (PAAD), pheochromocytoma and paraganglioma (PCPG), and uveal melanoma (UVM), and a protective factor of OS in THYM (Fig. 2B). Results for DSS, DFI, and PFI are shown in Fig. 2C–E. These results suggested that UBE2T expression had a strong prognostic power in different tumors, and high UBE2T expression frequently predicted a poor prognostic.

### Cancer-associated cellular processes regulated by UBE2T

**Fig. 3:**
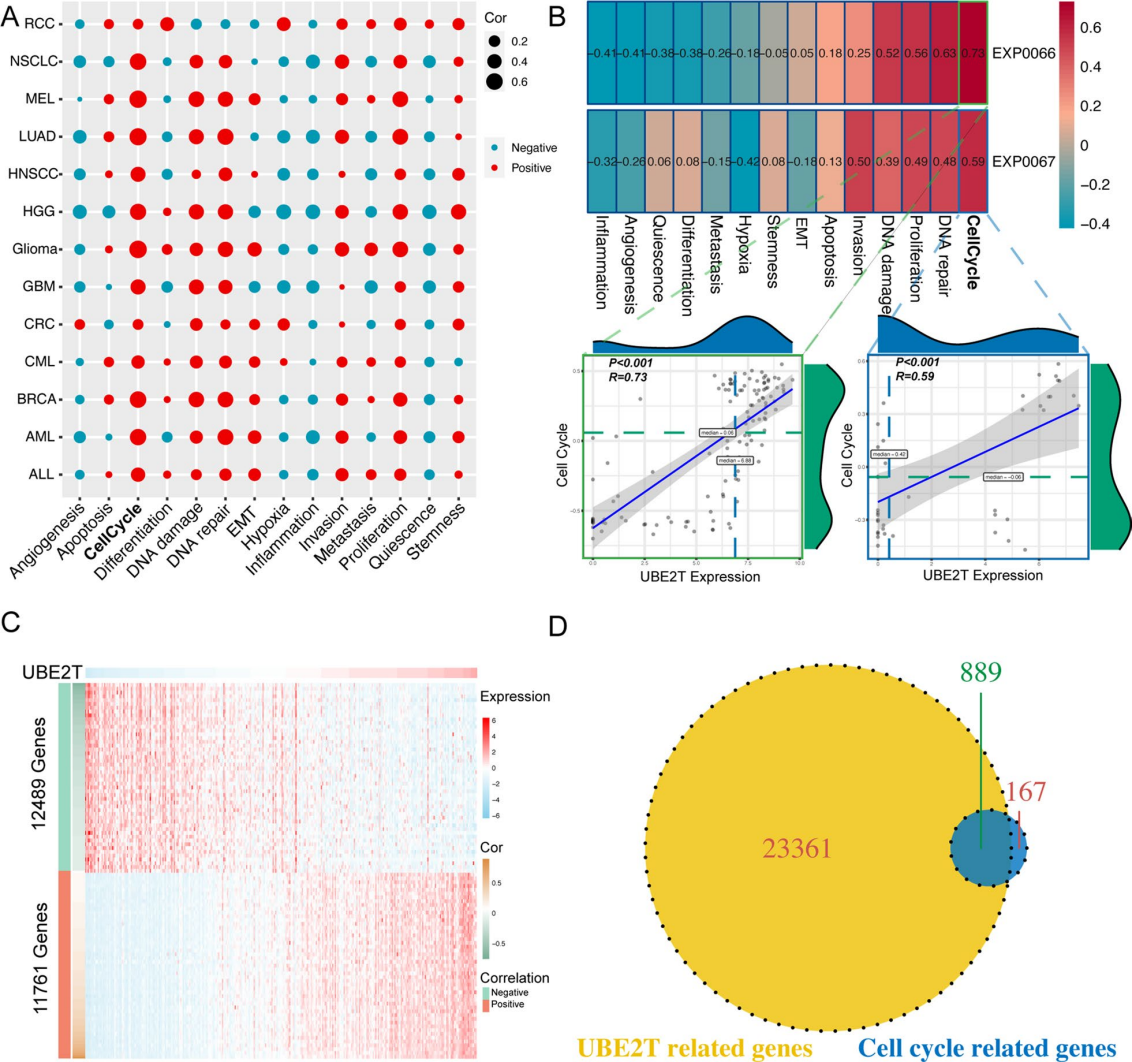
Determination of the association between UBE2T and functional states using single-cell sequencing datasets. **A** The association between the UBE2T gene and 14 functional states across 33 types of cancer, using single-cell sequencing datasets in the Cancer Single-cell State Atlas (CancerSEA) (http://biocc.hrbmu.edu.cn/CancerSEA/) database (The color of “Red” means positive correlation, while the color of “Blue” means negative correlation; The size of the circle represents the absolute value of the correlation coefficient). **B** The heatmap shows that the UBE2T gene is significantly associated with the functional states in two LUAD single-cell sequencing datasets. **C** The heatmap of the top 100 UBE2T-associated genes in LUAD. **D** The Venn diagram indicates the common elements between UBE2T-related genes and cell cycle genes

We next investigated whether UBE2T regulates some fundamental cancer-associated cellular processes by mining the CancerSEA database (http://biocc.hrbmu.edu.cn/CancerSEA/) [21]. UBE2T was associated with the cell cycle, DNA repair, proliferation, DNA damage, invasion, EMT, hypoxia, metastasis, differentiation, quiescence, angiogenesis, and inflammation in various cancer types, especially cell cycle and proliferation, among which cell cycle was prominently associated with many cancers (Fig. 3A). We focused on LUAD for the rest study due to its strong association with UBE2T. The expression of UBE2T was strongly associated with the cell cycle (r = 0.73, r = 0.59, p < 0.0001) in two single-cell RNA-Seq datasets on LUAD (Fig. 3B). Therefore, we decided to explore the clinical significance of UBE2T in LUAD by considering its regulatory roles in the cell cycle. By intersecting 1,056 cell cycle genes and 24,250 UBE2T-related genes in the TCGA-LUAD cohort (Fig. 3C), we obtained 889 UBE2T-related cell cycle genes (UrCCGs) (Fig. 3D).

### Identification and evaluation of subgroups of LUAD with WGCNA and LASSO Cox regression analyses

**Fig. 4:**
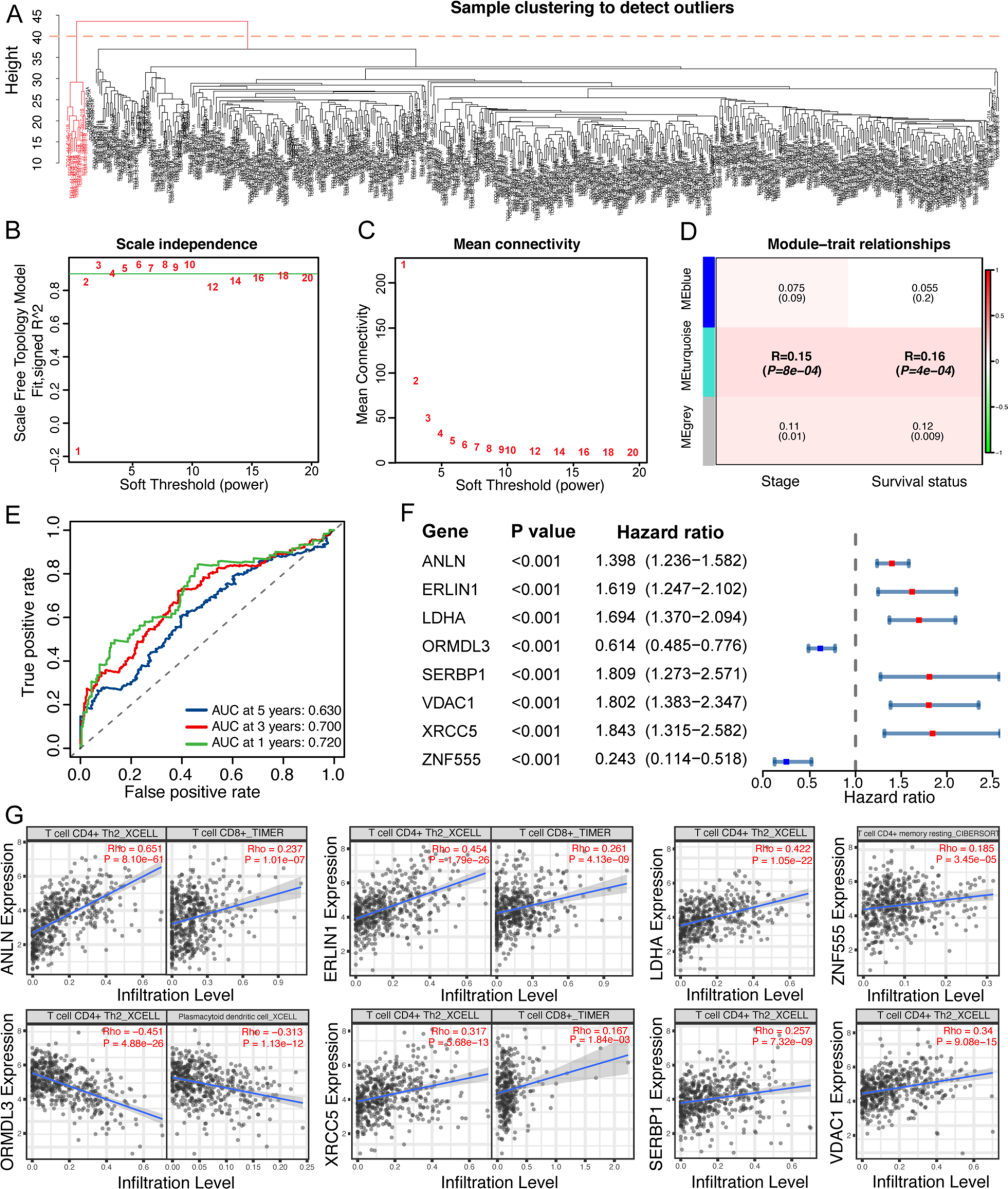
Development of a prognostic signature with the UBE2T-related cell cycle genes. **A** Weighted Gene Correlation Network Analysis (WGCNA) clustering of Samples. **B** The optimal soft-threshold (power) is estimated with the scale-free fit index. **C** The Mean connectivity for various soft thresholding powers. **D** Heatmap of the correlation between the module eigengenes and clinical traits of LUAD. We selected the MEturquoise-grade block for subsequent analysis. **E** The receiving operator characteristic (ROC) curves of 1, 3, and 5 years of the optimized 8-gene signature derived from LASSO regression analysis. **F** Forest plot shows the univariate Cox regression analysis of OS for the essential genes included in the prognostic signature. **G** Correlation between the expression levels of eight key genes and infiltration of CD + 4 T cells, dendritic cells, and CD + 8 T cells in LUAD tumors (TIMER2.0) (http://timer.comp-genomics.org/)

Cluster analysis on the samples was first performed to filter out possible outliers by presetting a height cut-off value of 40. After removing 11 samples, 503 samples were included to construct the WGCNA co-expression module of 889 UrCCGs (Fig. 4A). We correlated modules with clinical characteristics and found that the turquoise module was most significantly correlated with clinical stage and survival status (Fig. 4B–D). We then acquired 162 genes related to both stage and prognosis from the two modules and constructed an eight-gene signature through univariate prognostic analysis and LASSO Cox analysis. A risk score for each patient sample was calculated. The ROC showed that the risk score of the 8-gene signature had a fine predictive accuracy (Fig. 4E). Forest plots exhibited univariate analysis for genes included in the signature. (Fig. 4F). The Timer2.0 web tool (http://timer.comp-genomics.org/) revealed the association of the eight genes with critical immune cell infiltration (Fig. 4G). These findings demonstrate the important clinical significance of the UrCCGs.

### Identification and evaluation of subgroups of LUAD with consensus clustering

**Fig. 5:**
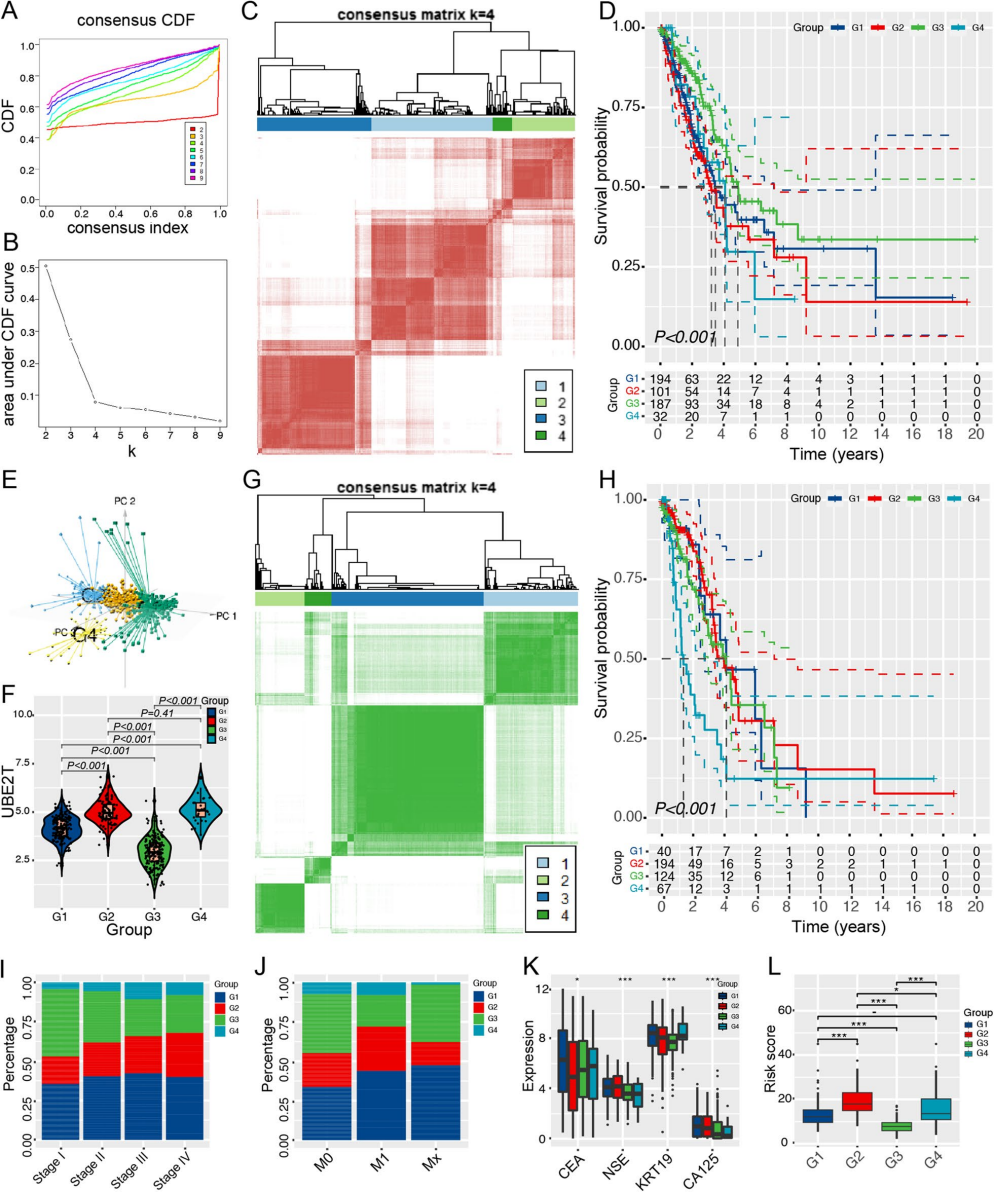
Identification and validation of potential UBE2T-related cell cycle gene (UrCCG) subtypes in LUAd using consensus clustering. **A**, **B** Cumulative distribution function curves (**A**) and delta area (**B**) of UrCCGs in TCGA-LUAD cohort. **C** Heatmap of sample clustering in TCGA-LUAD cohort. **D** Kaplan–Meier curves showing OS of UrCCG subtypes in TCGA-LUAD cohort. **E** The principal component analysis (PCA) map elucidates that UrCCG subtypes are independent. **F** Expression of UBE2T in four UrCCG subtypes. **G** Sample clustering heatmap of ICGC-LUAD cohort.(https://dcc.icgc.org/) **H** Kaplan–Meier curves showing OS of UrCCG subtypes in ICGC-LUAD cohort. **I**, **J** Distribution of G1-IG4 across stages (**I**) and metastasis (**J**) in TCGA-LUAD cohort. **K**, **L** Differential levels of tumor biomarkers (**K**) and risk score of the 8-gene signature (**L**) among the 4 subtypes in TCGA-LUAD cohorts

We analyzed the expression profiles of 889 UBE2T-related cell cycle genes from the TCGA database to construct consensus clustering using 514 LUAD samples. Based on the cumulative distribution function (Fig. 5A) and function delta area (Fig. 5B), we chose k = 4, where UBE2T-related cell cycle genes appeared to be stably clustered, and obtained four subtypes designated as G1-G4 (Fig. 5C). Kaplan–Meier curves showed that the G3 group was associated with the best prognosis, whereas G2 and G4 groups had the inferior survival probability (Fig. 5D). The principle component analysis (PCA) diagram showed that the four subtypes were independent of each other (Fig. 5E). Moreover, the expression of UBE2T was downregulated in the G3 subtype with a good prognosis when compared with G2 and G4 subtypes with poor prognosis (Fig. 5F). We also obtained consistent prediction results in the ICGC cohort, demonstrating the feasibility of our prognostic model (Fig. 5G, H). We further checked the distribution of subtypes in different stages and metastatic states. Consistently, The G3 subtype with favorable prognosis dominated clinical stage I (Fig. 5I) and M0 (Fig. 5J). Interestingly, we also found that G3 subgroups had lower levels of cancer biomarkers than G2 and G4 subgroups (Fig. 5K). The prognostic signature-derived risk score was lower in the G3 group than that in G2 and G4 groups (Fig. 5L). These results suggested that UrCCG subtypes could be used to evaluate the degree of malignancy, metastasis, and prognosis of LUAD patients.

### Different tumor-associated states among the four UrCCG subtypes

**Fig. 6:**
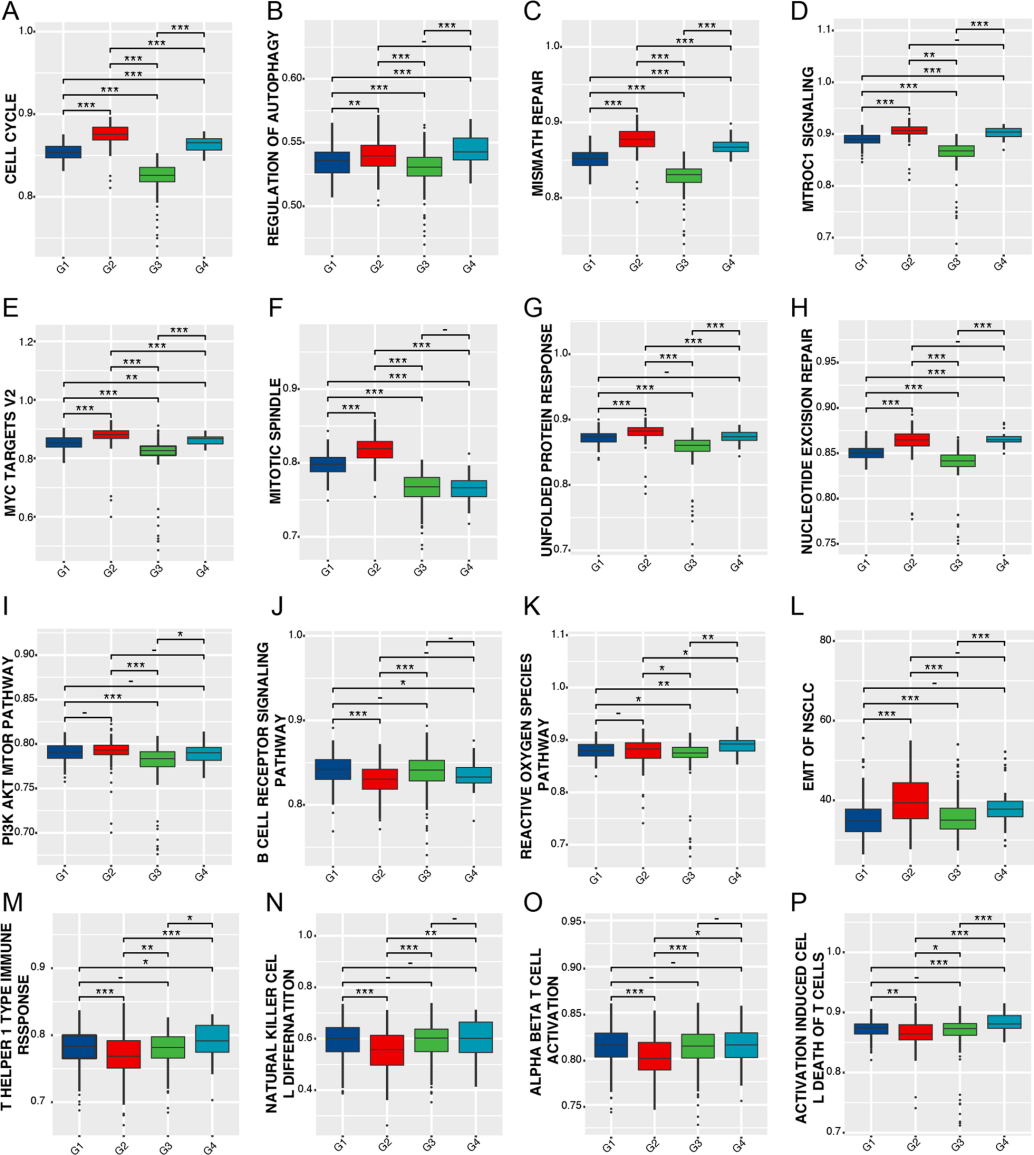
Gene Set Variation Analysis (GSVA) compares cellular, molecular, and immune characteristics of the four UrCCG subtypes based on KEGG, HALLMARK, or GO signatures. **A**–**P** Enrichment scores of the cell cycle (**A**), regulation of autophagy (**B**), mismatch repair (**C**), MTORC1 signaling (**D**), MYC-targets V2 (**E**), reactive oxygen species pathway (**F**), unfolded protein response (G), nucleotide expression repair (**H**), PI3K/AKT/mTOR (**I**), B cell receptor pathway (**J**), p53 pathway (**K**), EMT (**L**), alpha–beta T (αβT)-cell activation (**M**), natural killer cell differentiation (**N**), T helper 1 type immune response (**O**), and activation-induced cell death of T cells (**P**). *p < 0.05, **p < 0.01, and ***p < 0.001

We further interrogated the molecular and cellular characteristics underlying different subtypes using GSVA (http://www.gsea-msigdb.org/gsea/index.jsp). Compared with the G3 group, G2 and G4 groups had higher enrichment scores on tumor-related signaling pathways, including cell cycle, autophagy, mismatch repair, and MTORC1 signaling, but lower scores on the B cell receptor and p53 signaling pathway (Fig. 6A–K). Moreover, using the previously published EMT signature for NSCLC, we found that the EMT score was significantly higher in G2 and G4 subtypes with poor prognosis than in G3 subtypes with favorable prognosis (Fig. 6L). Interestingly, immunoenrichment results showed that G3 and G4 subtypes had higher levels of T helper 1 type immune response, natural killer cell differentiation, alpha–beta T (αβT)-cell activation, and activation-induced cell death of T cells (Fig. 6M–P).

### Association of UrCCG subtypes with tumor immune microenvironment

**Fig. 7:**
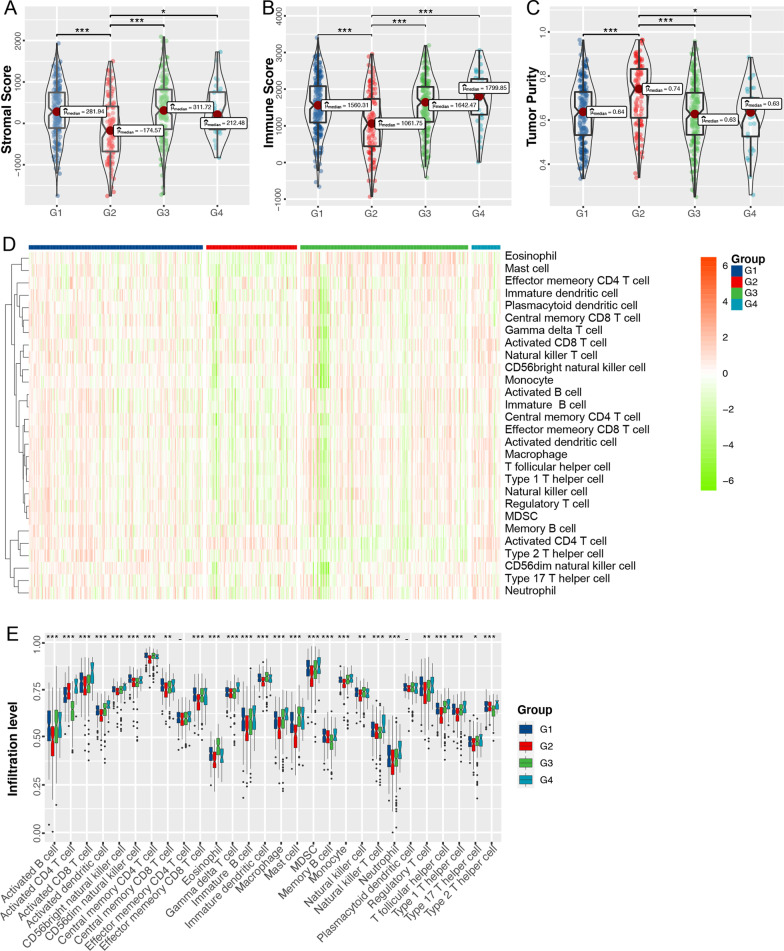
Investigation of differences in tumor immune cell infiltration among the four UrCCG subtypes. **A**–**C** ESTIMATE (Estimation of Stromal and Immune cells in Malignant Tumor tissues using Expression data) (https://bioinformatics.mdanderson.org/estimate/disease.html)method was used to calculate the stromal score (**A**), immune score (**B**), and tumor purity (**C**) of single samples in the TCGA-LUAD cohort, which were compared among G1–G4 subtypes (Kruskal–Wallis test). **D** Heatmap of enrichment scores of 28 immune cell signatures in TCGA-LUAD cohort sorted by UrCCG subtypes. E. Comparison of the cellular composition of 28 immune infiltrates among G1-G4 subtypes. *p < 0.05, **p < 0.01, and ***p < 0.001

Solid tumor tissue includes malignant cells, normal epithelial, stromal cells, immune cells, and vascular cells. The ssGSEA was performed on LUAD samples to analyze the differences in immune cell infiltration and tumor purity among different UrCCG subtypes by ESTIMATE (https://bioinformatics.mdanderson.org/estimate/disease.html) [26]. G3 and G4 showed higher immune infiltration (Fig. 7A, B) but lower tumor purity than G2 (Fig. 7C). The same analyses were also performed with the ICGC database (Additional file 2: Fig. S6A-C). We further explored the immune cell components in the four subtypes by scoring 28 previously reported signature genes in TCGA cohorts using ssGSEA. The enrichment of immune cell infiltrates significantly differed among the subtypes. Patients with G3 and G4 subtypes had more immune cell infiltration than those with G2 subtype (Fig. 7D, E). The ability of the UrCCG types to discriminate the levels of immune cell infiltration was also validated in the ICGC database (Additional file 2: Fig. S6D, E). These results suggest an association between UrCCG subtypes and immunity.

### The association between UrCCG subtype and immunotherapy sensitivity

**Fig. 8:**
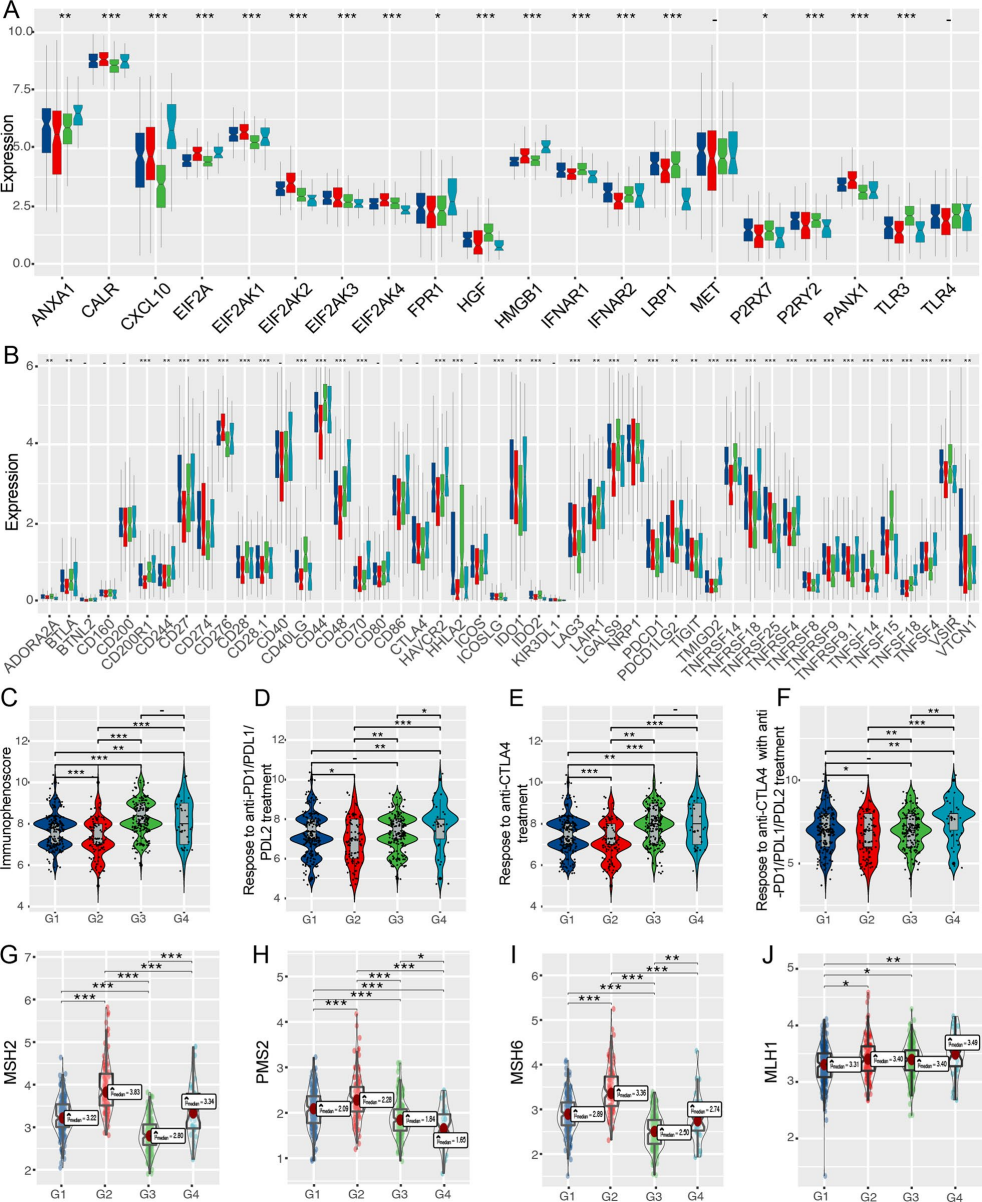
Association of UrCCG subtypes and immune checkpoints (ICPs), immunogenic cell death modulators (ICDs), immunophenoscore (IPS), and mismatch repair (MMR) genes in TCGA-LUAD cohort. **A**, **B** Differential expression of ICPs (**A**) and ICDs (**B**) among the G1-G4 subtypes. C-F. Determination of IPS (**C**), possibility of responding to anti-PD1/PDL1/PDL2 (**D**), anti-CTLA4 (**E**), and the combination (**F**) for G1-G4 subtypes. G-J. Expression levels of MMR genes for G1-G4 subtypes, including MSH2 (**G**), PMS2 (**H**), MSH6 (**I**), MLH1 (**J**). *p < 0.05, **p < 0.01, and ***p < 0.001

Given the importance of immune checkpoints (ICPs) [28] and immunogenic cell death (ICD) [29] modulators in tumor immunity, we examined the expression of ICPs and ICDs in different subtypes. Forty-seven ICPs-related genes were detected in TCGA-LUAD cohorts, of which 39 (83%) genes in the TCGA cohort were differentially expressed among the UrCCG subtypes (Fig. 8A). BTLA, CD160, CD200 CD200R1, CD244, CD27, CD28, CD28.1 CD40, CD44, CD48, CD80, CD86, HAVCR2, LAIR1, LGALS9, TNFRSF14, TNFRSF4, TNFSF18, and VTCN1 were significantly upregulated in G3, G4 subtypes compared to G2 in LUAD. Moreover, 26 (92.9%) ICD genes were differentially expressed among the subtypes (Fig. 8B). For instance, ANXA1, CXCL10, FPR1, and HMGB1 were significantly elevated in G4 subtypes compared to other subtypes.

Finally, we used immunophenoscore (IPS) for LUAD patients from The Cancer Immunome Atlas (TCIA, https://tcia.at/home) to determine the sensitivity to immune checkpoint inhibitors for the four subgroups, the most comprehensive immune determinant to date [27]. The G3 and G4 subgroups possessed significantly higher IPS than the G2 subgroups (Fig. 8C). Based on IPS, G3 and G4 subgroups tended to respond to either anti-PD1/PDL1/PDL2 or anti-CTLA4 antibodies or the combination of both agents (Fig. 8D–F). Besides, the expression levels of MSH2, MSH6, and PMS2 were significantly enhanced in patients with G2 subtypes compared to patients with G3 and G4 subtypes (Fig. 8G–J). Collectively, these results suggest that patients with G3 or G4 but not G2 subtypes may benefit from immunotherapy.

## Discussion

To the best of our knowledge, this was the first Pan-cancer study of UBE2T. By analyzing multi-omic databases, we found that the mRNA expression of UBE2T was upregulated in most tumors. High protein expression levels of UBE2T were validated in LUAD, UCEC, RCC, and OV. Survival analysis showed that UBE2T was associated with poor prognosis in most studied tumors. UBE2T has been implicated in the different tumor types. For instance, the expression level of UBE2T was positively correlated with the overall survival in HCC [7]. Survival association with UBE2T was also demonstrated in breast, osteosarcoma, nasopharyngeal carcinoma, prostate cancer, and esophageal cancer [6, 9, 12–15].

By analyzing single-cell RNA sequencing data of Pan-cancer, we found that UBE2T was associated with the most fundamental tumor-associated cellular events, including cell cycle, DNA repair, EMT, proliferation, and stemness. Before the advent of single-cell sequencing technology, the gene expression profiling of tumor samples could only be generated by the bulk sequencing method. Since malignant solid tumor tissues include tumor cells and normal stromal cells [30], some critical information about tumor cells may be overwhelmed by the stromal cells and immune cells. In the current study, we excluded the interference of other nontumor cells by utilizing the gene expression data from single-cell sequencing. Our findings are in lines with the previous study. For example, UBE2T was shown to upregulate EMT in renal cell carcinoma [31] and NSCLC cancer [10]. Guo et al. found that UBE2T regulated the proliferation and apoptosis of HCC through Wnt/β-Catenin and PI3K/Akt mTOR pathways [32]. Moreover, UBE2T could activate the Wnt signaling pathway to enhance the stemness of HCC cancer stem cells [8]. Mechanistic investigations further validate the oncogenic role of UBE2T. In HCC, UBE2T could promote the growth of tumor cells by facilitating the disintegration of p53 protein [7]. UBE2T mediated Mule ubiquitination and degradation, thereby accelerating tumor invasion in HCC [8]. In breast cancer, UBE2T promoted tumorigenesis via the ubiquitin-mediated degradation of BRCA1 [9]. A recent study revealed that UBE2T facilitated ubiquitin-dependent degradation of RACK1, a key scaffold protein stabilizing the β-catenin destruction complex in gastric cancer, leading to accumulation of β-Catenin and activation of the Wnt/pathway [11].

Single-cell data analysis indicated that the cell cycle is consistently and robustly associated with UBE2T across the different cancer types. Despite the complexity and uniqueness of each cancer, there were a limited number of shared “mission-critical” events promoting the uncontrolled expansion and invasion of tumor cells and their offspring. Among them, the cell cycle is the basis of all other phenotypes of tumor cells playing an indispensable role [33, 34]. Cell cycle dysregulation, implicated in malignant transformation and tumor progression, occurs in more than 90% of lung cancers, partially due to the aberrant activity of Cyclin-dependent kinases (CDK)–cyclin–RB pathways CDK4 and CDK6 can form complex with D-type cyclins, sequentially phosphorylate the Rb tumor suppressor protein, release E2F1, and thereby facilitate cell cycle progression Inhibiting CDK4/6 impairs cell cycle progression, suppresses tumor cell proliferation, and induces senescence. Several highly specific CDK4/6 inhibitors (Palbociclib, Ribociclib, and Abemaciclib), approved by the FDA for advanced metastatic breast cancer, showed great antitumor effects in preclinical lung cancer models [35] and have also been applied in clinical trials of lung cancer [36].

Given the critical association of UBE2T with cell cycle, combining UBE2T and its regulation on cell cycle may better predict cancer prognosis than single genes. We identified a risk signature of 8 UrCCGs with high accuracy in predicting the prognosis of LUAD. Several attempts to build prognostic signatures with cell cycle progression (CCP) genes have been reported [37–40]. Prognostic signatures derived from predetermined CCP genes, named CCP score, were developed and validated in prostate cancer [39, 40]. The predictive power of the CCP gene-derived signatures was also confirmed in early-stage lung adenocarcinoma [37, 38]. Furthermore, Chen and colleagues recently developed a risk-predicting signature with eight immunity-related cell cycle genes [41]. These findings indicate the reliability of predicting prognosis with CCP signatures in cancer. Consistently, our study identified and validated the prognostic discrimination of oncogene-associated CCP genes. Our composite clinical and UrCCG signature showed decent prognostic accuracy, which is comparable to other existing prognostic signatures [41–43]. Interestingly, these UrCCGs in the risk signature had a strong correlation with CD4 + Th2 cell, CD8 + T cell, DC, and CD4 + T memory resting cell. The association between the eight genes and tumorigenesis has been reported in previous publications [15, 44–51]. For example, Liu et al. proved that ANLN regulated the proliferation of colorectal cancer cells through PI3K/Akt and MAPK pathways, suggesting that ANLN might be a new target for the treatment of colorectal cancer [15]. These results suggest that these UrCCGs are also potential targets in cancer management.

Furthermore, based on the differential expression patterns of UrCCGs, CC analysis divided the TCGA-LUAD cohorts into G1 to G4 subtypes, with significantly different survival. G3 subtype patients had better survival than other subtypes. The four subtypes exhibited different molecular, cellular, and clinical characteristics. Consistently, the G3 subtype with better survival had lower expression levels of UBE2T than the G2 and G4 subtypes. G3 subtype patients predominated in clinical stage I and M0 subgroups, while the fraction of G2 and G2 subtypes increased with advanced stage and metastatic status. The subtypes were also associated with tumor biomarkers such as NSE, KRT19, and CA125. Overall, the survival status predicted by UrCCGs agrees with the traditional TNM stage, tumor metastasis status, and tumor biomarkers.

Regarding the molecular and cellular characteristics, compared with the G3 group, G2 and G4 groups had higher enrichment scores on tumor-related signaling pathways, including cell cycle, regulation of autophagy, mismatch repair, MTORC1 signaling, MYC-targets V2, unfolded protein response, nucleotide expression repair, PI3K/AKT/mTOR, reactive oxygen species pathway, but lower scores on B cell receptor pathway and p53 pathway. EMT played an essential role in tumor metastasis [52]. With a new EMT signature in NSCLC and weighting coefficients [42], we demonstrated that the EMT score in G2 and G4 subtypes with poor prognosis was higher than that in the G3 subtype. These results indicated substantial differences in tumor-associated phenotypes among the four subtypes. Enrichment of malignant characteristics might help explain the poor prognosis in patients with G2 and G4 subtypes.

We analyzed the relationship between different subtypes and immunity. Intriguingly, tumors with G3 and G4 subtypes had higher levels of immune cell infiltration than tumors with G2 subtype. First, ESTIMATE analysis indicated that patients with G3 and G4 subtypes had higher stromal-score and immune-score but lower tumor purity than G2 type. The ssGSEA was adopted to calculate stromal score and immune score for each tumor sample with “stromal gene signature” and “immune gene signature,” respectively [26]. Second, Augmented T helper 1 type immune response, natural killer cell differentiation, activation of αβT cells, and activation induced cell death of T cells also manifested an immunogenic tumor environment in G2 and G4 subtypes. Generally, the activation of αβT cells relies on antigen presented by the major histocompatibility complex (MHC) proteins. Upon antigen recognition, αβT cells transit into cytotoxic effector cells or secret cytokines [53]. AICD is programmed cell death in activated T cells depending on the Fas receptors (Fas, CD95)/Fas ligands (FasL, CD95 ligand) pathway. The immune system’s hyperactivation or constitutive activation can induce T-cell exhaustion and AICD in T- and B-cells. Therefore, the increased T cell AICD may reflect the overactivation of antitumor immunity in the G3 and G4 subgroups [54].

Moreover, with a published algorithm [27], we comprehensively compare the 28 different types of infiltrated immune cells among the four subtypes comprising memory cells, cytotoxic cells, and immunosuppressive cells. Overall, higher levels of immune cell infiltration were observed in G3 and G4 compared with G2 subtypes. More importantly, the fraction of activated CD8 T cells, central memory CD8 T cells, and effector memory CD8 T cells were significantly enhanced in the former. These results suggest that G3 and G4 subtypes belonged to the “hot” immune tumor and the G2 subtype belonged to the “cold” immune tumor. In 2009, Camus et al. originally delineated three major immune coordination patterns (hot, altered, and cold) in the primary CRC [55]. Nowadays, the “hot” and “cold” immune patterns are used to indicate T cell-infiltrated, inflamed but non-infiltrated, non-inflamed tumors [25]. In general, the “hot” immune pattern of tumors is more likely to benefit from immunotherapy. Furthermore, we found that most of the ICP and ICD genes were differentially expressed in the four subgroups. Fortunately, immunophenoscore was developed to comprehensively quantify tumor immunogenicity by integrating antigen processing (MHC), checkpoints, immunomodulators, effector cells (activated CD8, activated CD4, Tem CD4, Tem CD8, and suppressor cells (Treg and MDSC). Immunophenoscore has been reported to robustly predict response to anti-CTLA and anti-PD1 antibodies [27]. With the utilization of IPS, we detected significantly higher IPS in G3 and G4 subgroups than in the G2 group, and the former showed the increased possibility of responding to PD1/PDL1/PDL2 inhibitor, CTLA4 inhibitor, and the combination of ICIs. Consistently, G3 and G4 subtypes exhibited lower levels of MMR genes (MSH2, PMS2, and MSH6) than G3 types. MMR is known to be a powerful adjunct marker for predicting immunotherapy. Deficiency in MMR genes is also considered an indicator of response to ICIs [56]. Collectively, the UBE2T-related cell cycle subtypes may inform clinicians for decision-making. Despite the merits of the current study, limitations are unavoidable. The results presented in this study were mainly based on bioinformatic analyses. Validating the UBET2 using in-house clinical samples could great strengthen our findings.

## Conclusion

In conclusion, UBE2T is a critical oncogene. Moreover, UBE2T-related cell cycle genes could separate the LUAD cohort into four subgroups with significant differences in survival, immune cell infiltration, and immunotherapy sensitivity. Our findings demonstrated that UBE2T could be a therapeutic target, as well as a predictor of survival and immunotherapy in cancer treatment.

### Summary of public databases

1. The TCGA database. https://portal.gdc.cancer.gov/. Accessed 20 Nov 2021.

2. The Gencode (GENCODE.v32). https://ftp.ebi.ac.uk/pub/databases/gencode/Gencode_human/. Accessed 11 Nov 2021.

3. The Gene Expression Profiling Interactive Analysis. http://gepia.cancer-pku.cn/. Accessed 15 Nov 2021.

4. The Genotype-Tissue Expression database. https://www.genome.gov/Funded-Programs-Projects/Genotype-Tissue-Expression-Project. Accessed 11 Sep 2021.

5. The UALCAN portal. http://ualcan.path.uab.edu/analysis-prot.html. Accessed 10 Nov 2021.

6. The Human Protein Atlas. https://www.proteinatlas.org/. Accessed 23 Oct 2021.

7. The Cancer Single-cell State Atlas. http://biocc.hrbmu.edu.cn/CancerSEA/. Accessed 5 Aug 2021.

8. The tumor immune estimation resource database. http://timer.comp-genomics.org/. Accessed 27 Nov 2021.

9. The ICGC cohort. https://dcc.icgc.org/. Accessed 7 Nov 2021.

10. The Cancer Immunome Atlas. https://tcia.at/home. Accessed 21 May 2021.

## Supplementary Information

**Additional file 1: Figure S1.** The flowchart of the study.

**Additional file 2: Figure S2.** Association between UBE2T gene expression and overall survival (OS) of 33 different types of tumors in TCGA database. A-K. Significant association between UBE2T and OS of ACC (A), BRCA (B), KIRC (C), KIRP (D), LGG (E), LIHC (F), LUAD (G), MESO (H), OV (I), STAD (J), and THYM (K).

**Additional file 3: Figure S3.** Association between UBE2T gene expression and disease-specific survival (DSS) of 33 different types of tumors in TCGA database. A-J. The significant association between UBE2T and DSS of ACC (A), COAD (B), KICH (C), KIRC (D), KIRP (E), LGG (F), LUAD (G), MESO (H), OV (I), and PCPG (J).

**Additional file 4: Figure S4.** Association between UBE2T gene expression and disease free interval (DFI) of 33 different types of tumors in TCGA database. A-J. Significant association between UBE2T and DFI of ACC (A), BRCA (B), KIRP (C), LIHC (D), LUAD (E), MESO (F), PAAD (G), PRAD (H), STAD (I), and THCA (J).

**Additional file 5: Figure S5.** Association between UBE2T gene expression and progression-free interval (PFI) of 33 different types of tumors in TCGA database. A-K. The significant association between UBE2T and OS of ACC (A), KICH (B), KIRC (C), KIRP (D), LGG (E), LIHC (F), LUAD (G), MESO (H), PARD (I), STAD (J), and UVM (K).

**Additional file 6: Figure S6.** Validation of discrepancies in tumor immune environment among the four UrCCG subtypes in ICGC-LUAD cohorts. A-C. Differences in the stromal score (A), immune score (B), and tumor purity (C) (Kruskal–Wallis test). D. Heatmap of 28 types of infiltrating immune cells. E. The fractions of 28 infiltrating immune cells were compared among G1-G4 subtypes.

**Additional file 7.** Detailed methods for the construction of risk signature with UrCCGs, discovery and validation of the subtypes by the UBE2T-related cell cycle genes, and investigation of different tumor-associated cellular and molecular states and immunotherapy sensitivity among the four UrCCG subtypes.

## Data Availability

The original contributions presented in the study are included in the article/Supplementary Material. Further inquiries can be directed to the corresponding authors.

## Abbreviations

UBE2T: Ubiquitin-conjugating enzyme E2 T
ACC: Adrenocortical carcinoma
BLCA: Bladder urothelial carcinoma
BRCA: Breast invasive carcinoma
CESC: Cervical squamous cell carcinoma and endocervical adenocarcinoma
CHOL: Cholangiocarcinoma
COAD: Colon adenocarcinoma
DLBC: Lymphoid neoplasm diffuse large b-cell lymphoma
ESCA: Esophageal carcinoma
GBM: Glioblastoma multiforme
HNSC: Head and neck squamous cell carcinoma
KICH: Kidney chromophobe
KIRC: Kidney renal clear cell carcinoma
KIRP: Kidney renal papillary cell carcinoma
LAML: Acute myeloid leukemia
LGG: Brain lower grade glioma
LIHC: Liver hepatocellular carcinoma
LUAD: Lung adenocarcinoma
LUSC: Lung squamous cell carcinoma
MESO: Mesothelioma
OV: Ovarian serous cystadenocarcinoma
PAAD: Pancreatic adenocarcinoma
PCPG: Pheochromocytoma and paraganglioma
PRAD: Prostate adenocarcinoma
READ: Rectum adenocarcinoma
SARC: Sarcoma
SKCM: Skin cutaneous melanoma
STAD: Stomach adenocarcinoma
TGCT: Testicular germ cell tumors
THYM: Thymoma
THCA: Thyroid carcinoma
UCS: Uterine carcinosarcoma
UCEC: Uterine corpus endometrial carcinoma
UVM: Uveal melanoma
OS: Overall survival
DSS: Disease-specific survival
DFI: Disease-free interval
PFI: Progression free interval
UrCCGs: UBE2T-related cell cycle genes
IPS: Immunophenoscore
