# Supplementary figures and images for: Pan-cancer analysis of UBE2T with a focus on prognostic and immunological roles in lung adenocarcinoma
Source: Respir Res. 2022 Nov 10;23:306. doi: 10.1186/s12931-022-02226-z (PMC9650835; doi:10.1186/s12931-022-02226-z)

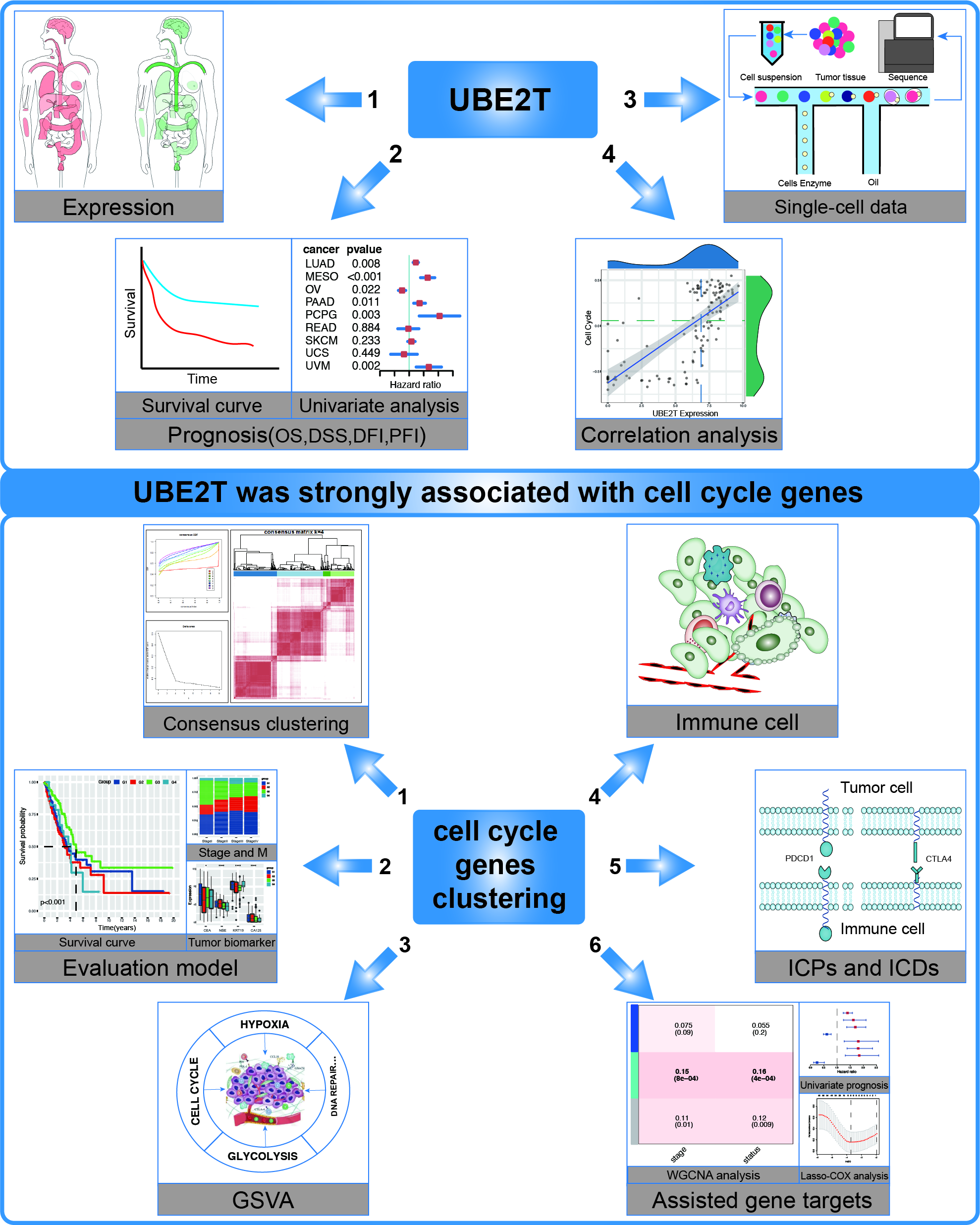

Supplement: Supplementary file 1 — Additional file 1: Figure S1. The flowchart of the study. [file 12931_2022_2226_MOESM1_ESM.tif]

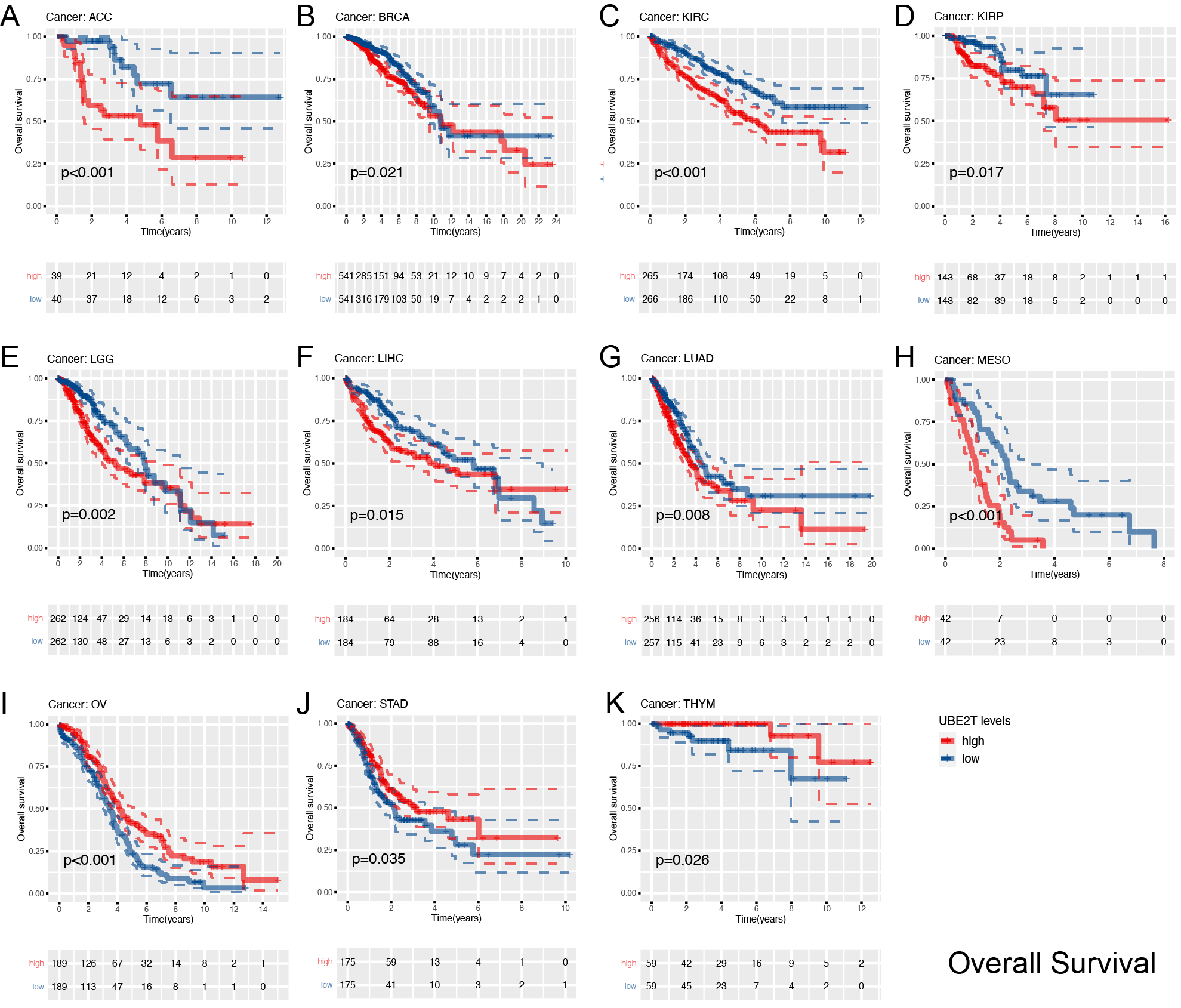

Supplement: Supplementary file 2 — Additional file 2: Figure S2. Association between UBE2T gene expression and overall survival (OS) of 33 different types of tumors in TCGA database. A-K. Significant association between UBE2T and OS of ACC (A), BRCA (B), KIRC (C), KIRP (D), LGG (E), LIHC (F), LUAD (G), MESO (H), OV (I), STAD (J), and THYM (K). [file 12931_2022_2226_MOESM2_ESM.tif]

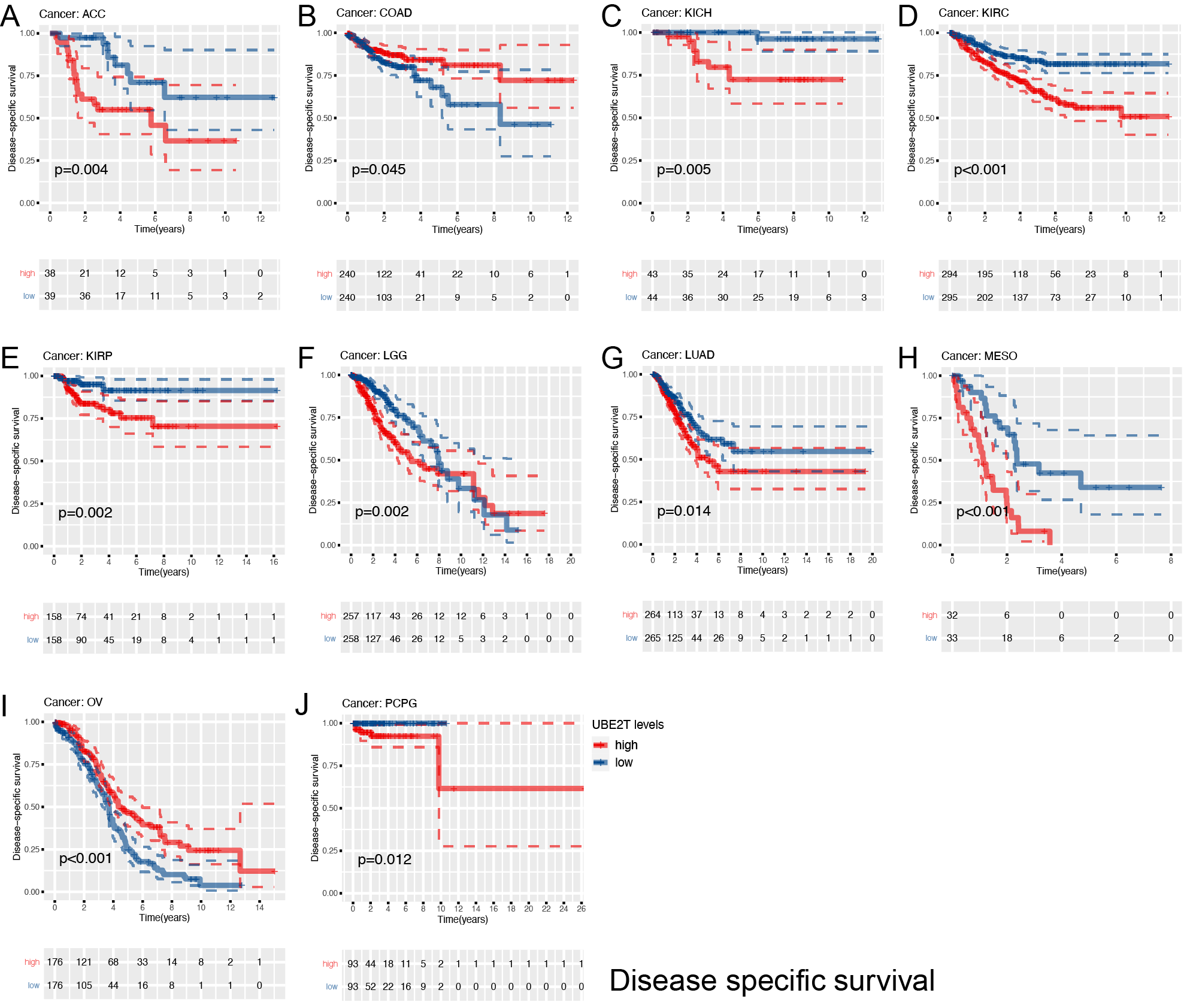

Supplement: Supplementary file 3 — Additional file 3: Figure S3. Association between UBE2T gene expression and disease-specific survival (DSS) of 33 different types of tumors in TCGA database. A-J. The significant association between UBE2T and DSS of ACC (A), COAD (B), KICH (C), KIRC (D), KIRP (E), LGG (F), LUAD (G), MESO (H), OV (I), and PCPG (J). [file 12931_2022_2226_MOESM3_ESM.tif]

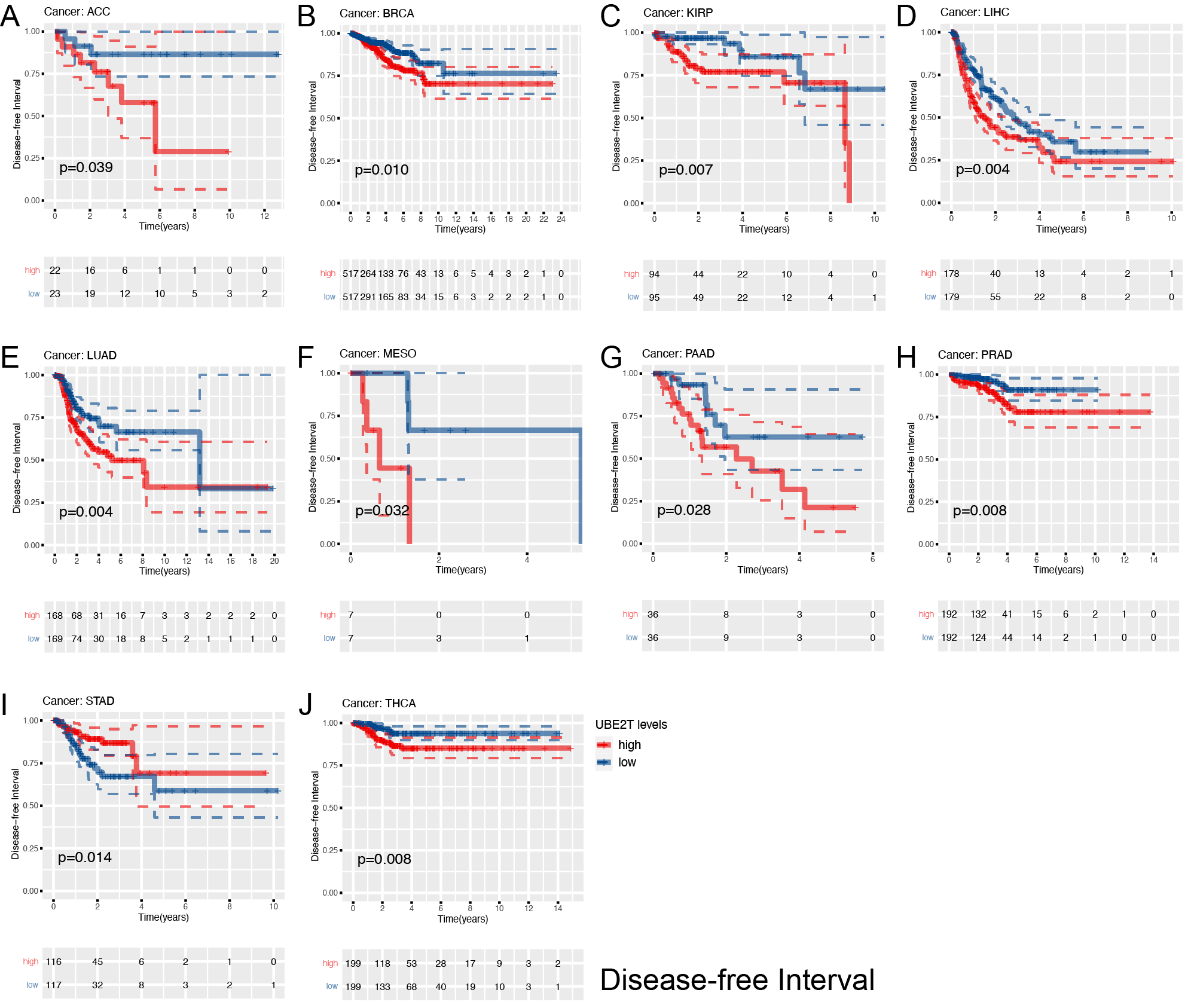

Supplement: Supplementary file 4 — Additional file 4: Figure S4. Association between UBE2T gene expression and disease free interval (DFI) of 33 different types of tumors in TCGA database. A-J. Significant association between UBE2T and DFI of ACC (A), BRCA (B), KIRP (C), LIHC (D), LUAD (E), MESO (F), PAAD (G), PRAD (H), STAD (I), and THCA (J). [file 12931_2022_2226_MOESM4_ESM.tif]

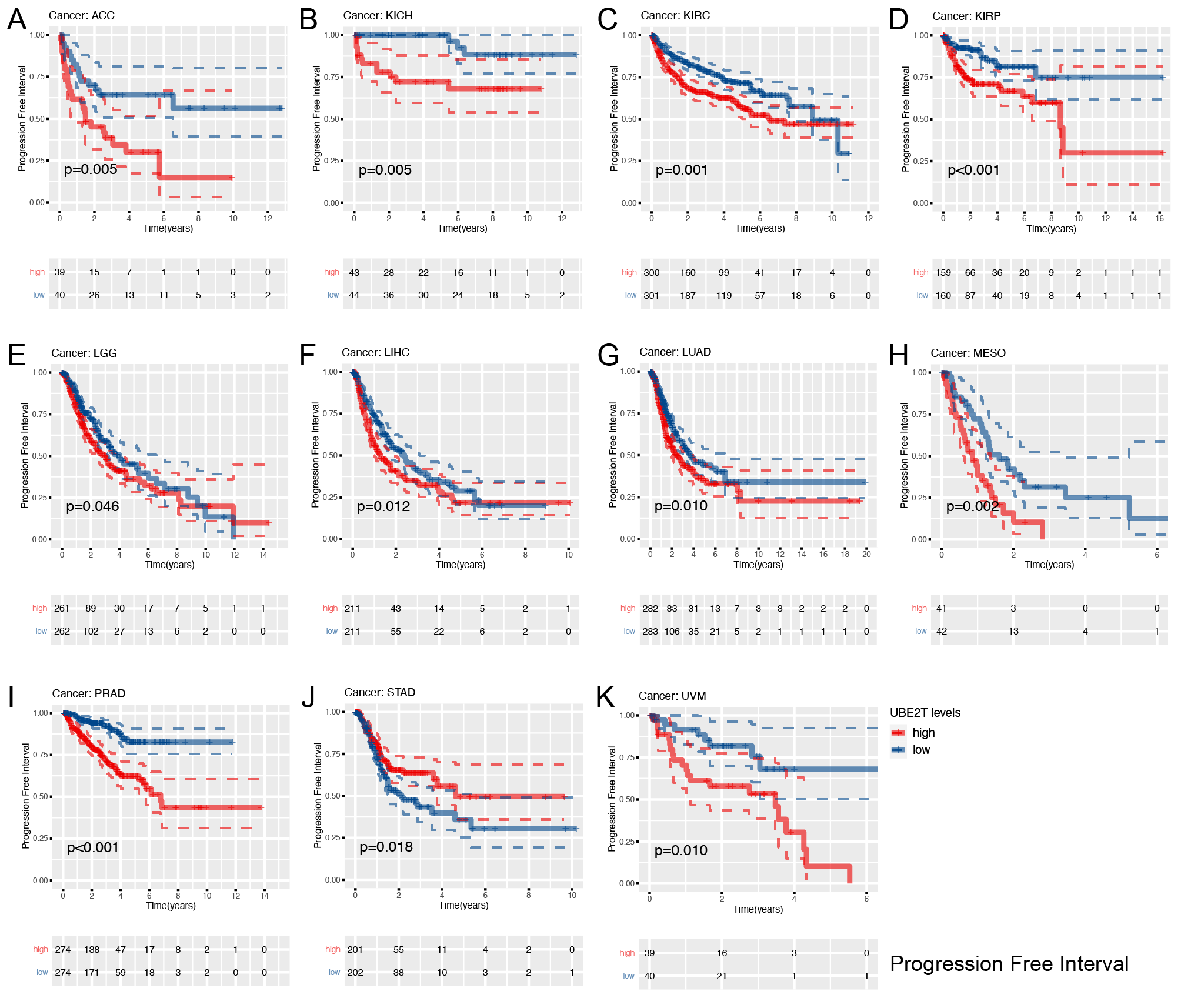

Supplement: Supplementary file 5 — Additional file 5: Figure S5. Association between UBE2T gene expression and progression-free interval (PFI) of 33 different types of tumors in TCGA database. A-K. The significant association between UBE2T and OS of ACC (A), KICH (B), KIRC (C), KIRP (D), LGG (E), LIHC (F), LUAD (G), MESO (H), PARD (I), STAD (J), and UVM (K). [file 12931_2022_2226_MOESM5_ESM.tif]

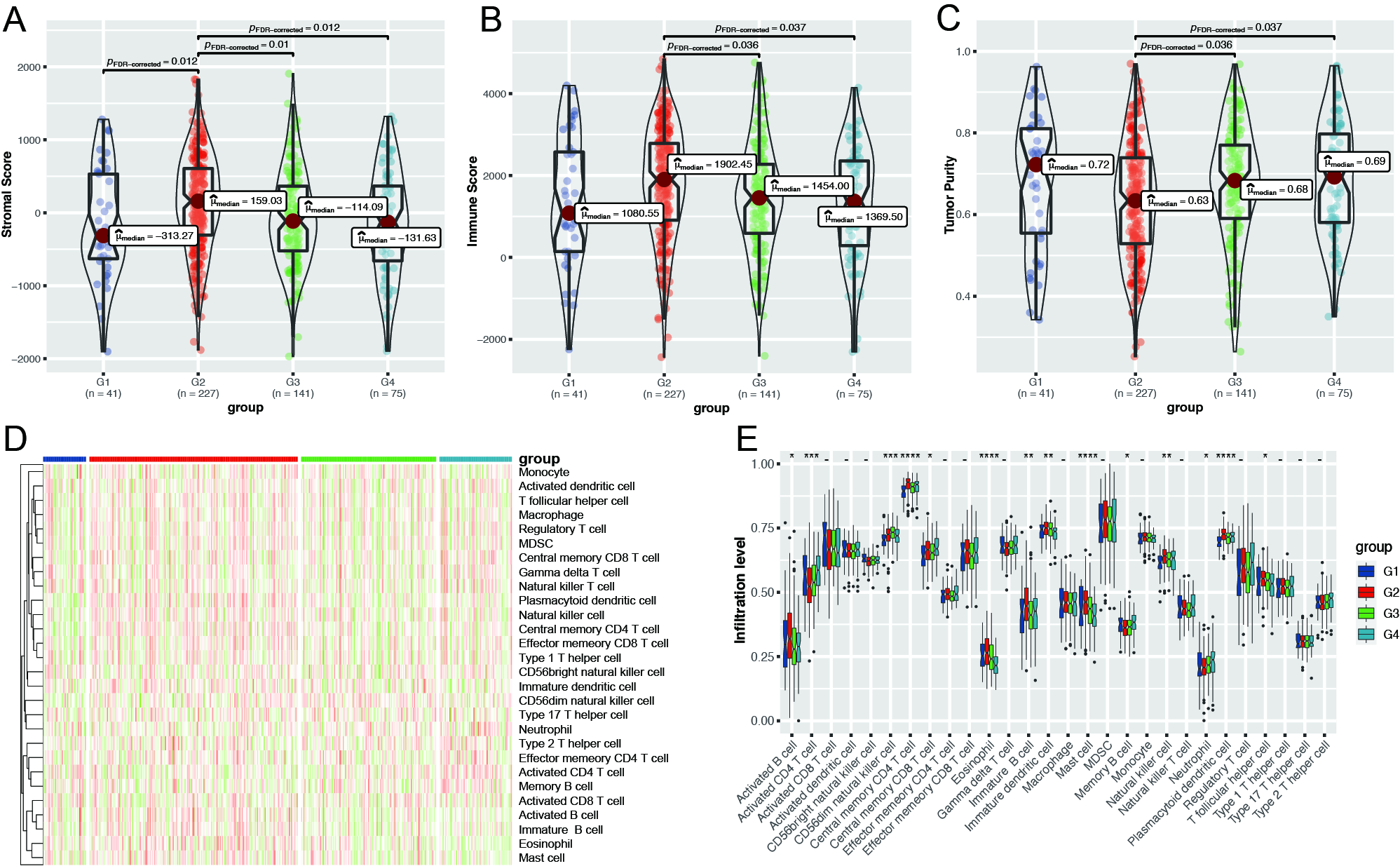

Supplement: Supplementary file 6 — Additional file 6: Figure S6. Validation of discrepancies in tumor immune environment among the four UrCCG subtypes in ICGC-LUAD cohorts. A-C. Differences in the stromal score (A), immune score (B), and tumor purity (C) (Kruskal–Wallis test). D. Heatmap of 28 types of infiltrating immune cells. E. The fractions of 28 infiltrating immune cells were compared among G1-G4 subtypes. [file 12931_2022_2226_MOESM6_ESM.tif]
