# Supplementary material for: Pan-cancer analysis of UBE2T with a focus on prognostic and immunological roles in lung adenocarcinoma
Source: Respir Res. 2022 Nov 10;23:306. doi: 10.1186/s12931-022-02226-z (PMC9650835; doi:10.1186/s12931-022-02226-z)
Supplement: Supplementary file 7 — Additional file 7. Detailed methods for the construction of risk signature with UrCCGs, discovery and validation of the subtypes by the UBE2T-related cell cycle genes, and investigation of different tumor-associated cellular and molecular states and immunotherapy sensitivity among the four UrCCG subtypes. [file 12931_2022_2226_MOESM7_ESM.zip › Additional file 7.docx]

**Construction of risk signature with UrCCGs**

Step 1: UBE2T-associated gene set

We determined genes related to UBE2T expression in the TCGA-LUAD by Pearson correlation analysis (P<0.05, |r>0.1|), and retrieved a list of 24,250 UBE2T-related genes.

Step 2: Cell cycle gene set

A total of 1,056 genes related to the cell cycle were downloaded from the CancerSEA database (http://biocc.hrbmu.edu.cn/CancerSEA/).

Step 3: Identification of UrCCGs

A Venn diagram using the “VennDiagram” package was used to identify genes shared by the above two gene sets (n=889), which were considered UBE2T-related cell cycle genes (UrCCGs).

Step 4: Weighted gene coexpression network analysis (WGCNA) of UrCCGs

The WGCNA was used to identify genes that were coexpressed with UrCCGs in the TCGA-LUAD. Module-trait relationships were evaluated based on the correlation between modules and traits by Pearson’s correlation test, and modules were considered significantly correlated when P ≤ 0.05. Two gene coexpression modules were found to be significantly associated with the stage (n=176) and survival status (n=388) traits. Totally, 162 overlapping genes were retrieved from the two gene coexpression modules.

Step 5: Construction of risk signature with UrCCGs

Univariate Cox regression analysis was applied to pick out 83 prognostic genes from 162 overlapping genes (P < 0.001). The prognostic genes were entered into LASSO Cox analysis was conducted to construct an optimal risk signature, with a 10-round cross-validation restricting overfitting. The correlations between the expression of genes in risk signature and immune cell infiltration, including CD8+ T cells, CD4 + T cells, and dendritic cells, were analyzed by the “Gene” module of the tumor immune estimation resource (TIMER2.0) database_8_ (<http://timer.comp-genomics.org/)>. The correlation between gene expression and immune infiltration was estimated by the Pearson correlation test.

**Discovery and validation of the subtypes by the UBE2T-related cell cycle genes**

Step 1: Identification of subgroups by UrCCGs

The ConsensusClusterPlus was used to cluster the TCGA-LUAD patients into independent groups with 889 UBE2T-related cell cycle genes. We conducted 500 bootstraps, and each subsample contained 80% of patients in the cohort. Cluster sets ranged from 2 to 9, and the optimal separation was determined by evaluating the consensus matrix and the consensus cumulative distribution function. As a result, the TCGA-LUAD patients were divided into four subgroups.

Step 2: Kaplan-Meier survival analysis of UrCCG subgroups

We performed the Kaplan-Meier survival analysis and log-rank test for the four subgroups using the R packages of survival and survminer.

Step 3: Validation in an independent LUAD cohort.

The subtypes by UrCCGs were validated in an independent ICGC-LUAD cohort (https://dcc.icgc.org/) with the same settings, i.e., International Cancer Genome Consortium (ICGC)-LUAD cohort (https://dcc.icgc.org/).

Step 4: Comparison of the distribution of stage, metastasis, cancer markers, and risk score in the four UrCCG subgroups

Results suggested that UrCCG subtypes could be used to evaluate the degree of malignancy, metastasis, and prognosis of LUAD patients.

**Different tumor-associated states and immunotherapy sensitivity among the four UrCCG subtypes**

Step 1: Evaluation of tumor-associated states among the four UrCCG subtypes

We further interrogated the molecular and cellular characteristics underlying the four UrCCG subtypes using GSVA. Gene sets represented different tumor-associated states (e.g., unfolded protein response and EMT) were retrieved from HALLMARK, KEGG pathway, and Gene Ontology databases. GSVA was used to compute the activity of indicated tumor-associated states for each tumor sample using corresponding gene sets. The single-sample Gene Set Enrichment Analysis (ssGSEA) algorithm of the R package “GSVA” was applied.

Step 2: Estimation of intratumoral immune infiltrates

First, the ESTIMATE algorithm was used to assess the differences in the stromal score, immune score, and tumor purity among the four UrCCG subtypes. Second, we downloaded metagene sets representing 28 immune cell subpopulations from TCIA (https://tcia. at/home) and ssGSEA were conducted to calculate the proportions of these immune cell subsets in each tumor sample.

Step 3: Gene expression levels of immune checkpoints (ICPs) and immunogenic cell death modulators (ICDs) were downloaded for the TCGA-LUAD patients (https://portal.gdc.cancer.gov/).

Step 4: immunophenoscore (IPS) of LUAD patients was downloaded from TCIA (https://tcia. at/home). The IPS is calculated based on four significant categories of tumor immunogenicity determinants, including effector cells, immunosuppressive cells, major histocompatibility complex (MHC) molecules (antigen processing), and checkpoints/immunomodulators. The IPS, ranging from 0 to 10, was calculated based on the z-score for the expression of related genes
